# Unveiling contextual influences of maternal satisfaction with labour care services in Nigeria: A qualitative inquiry

**DOI:** 10.4102/phcfm.v15i1.4173

**Published:** 2023-11-28

**Authors:** Titilope A. Awotunde, Mary Ani-Amponsah, Dolapo E. Ajala, Simeon A. Ojo, Thomas O. Adeleke, Olufemi T. Awotunde, Akintayo D. Olaolorun

**Affiliations:** 1 Department of Nursing, College of Health Sciences, Bowen University, Iwo, Nigeria; 2 Department of Maternal and Child Health, School of Nursing and Midwifery, University of Ghana, Legon, Ghana; 3 Department of Nursing, Faculty of Clinical Sciences, Bowen University Teaching Hospital, Ogbomoso, Nigeria; 4 Department of Family Medicine, College of Health Sciences, Bowen University, Iwo, Nigeria

**Keywords:** labour, maternal satisfaction, postnatal, labour care services, factors, qualitative inquiry, childbirth

## Abstract

**Background:**

In light of the rising global effort to lower maternal mortality rates, it is crucial for low- and middle-income countries with poor maternal indices to investigate the problem of maternal satisfaction and the key elements that affect it. To this effect, this study explored the experiences of postnatal women in relation to labour services and investigated the factors that contribute to their overall satisfaction.

**Aim:**

The study set out to explore factors influencing maternal satisfaction with labour care services in Ogbomoso, Oyo State, Nigeria. This study ultimately seeks to advance our understanding of this phenomenon to impact labour care and policy.

**Setting:**

The study was conducted among multiparous women who had their antenatal care and delivery in Ogbomoso, Oyo State, Nigeria.

**Methods:**

A qualitative study was performed using in-depth interviews among postnatal women.

**Results:**

The results revealed a number of variables that could affect the women’s satisfaction with labour care, including the choice of health facility, healthcare providers, environment of the facility, assurance of privacy, treating patients with dignity, provision of needed amenities and having a well-planned postnatal care assessment.

**Conclusion:**

The study revealed that the costs of care, the skill of the caregiver, the provision of confidential and dignified care, and the availability of supplies all have an impact on maternal satisfaction. Hospital administration should address these issues to enhance the experience of women and labour care services.

**Contribution:**

The study’s findings provide insights that will inform strategies to improve the quality of care being provided to parturients in Nigeria.

## Introduction

Labour and childbirth are regarded as periods of uncertainty and intense pain for pregnant women.^1,2^ Satisfaction with healthcare services rendered during labour reflects the dynamic and subjective perception of the extent to which a parturient experiences the positive perception of the care provided by the healthcare provider.^3,4^

Maternal satisfaction is one of the most frequently reported measures for quality of care that attracts urgent attention and highlights the multidimensional aspects of quality-of-care provision indicated in the World Health Organization’s (WHO) quality-of-care framework.^5,6^ Scholars across the globe have probed into what influences maternal satisfaction with maternity care services, attesting to the fact that this concept has gained widespread recognition in the area of maternal and child health research.^5,6,7^ Furthermore, the concept of maternal satisfaction is crucial to controlling and administering the quality of healthcare, which can inform service development and delivery as well as viability and sustainability.^8,9^

Sources of maternal dissatisfaction with labour care services include poor staff attitude, disrespectful maternal services, poor attention to women in labour, high cost of services and substandard facilities to mention but a few.^10,11,12,13,14,15^ These studies on maternal dissatisfaction with labour care services were linked to why women in Nigeria sometimes prefer the services of traditional birth attendants rather than modern health facilities. In line with this, exploring maternal satisfaction with labour care appears germane to monitoring and evaluating the quality of maternity care in Nigeria owing to the unacceptable maternal mortality rate of 814 (per 100 000 live births) according to the WHO.^16^

Thus, it is important for low- and middle-income countries (LMICs) with poor maternal indices to explore the issue of maternal satisfaction and the major factors that affect it in different contexts, at a time that there is increased global effort to reduce maternal mortality rates.^17^

Despite the high level of maternal satisfaction from studies on maternity care services across Nigeria, results from the reviewed literature seem not to be consistent and are inconclusive to determine the factors that influence satisfaction with labour care services in the country. A few quantitative studies conducted in south-west Nigeria focussed on satisfaction with labour care services.^13,18,19^ This study was conducted among postnatal women in Ogbomoso to qualitatively explore factors that influence maternal satisfaction with the aim of adding to the body of knowledge on this important issue and perhaps the findings can influence policy change. In order to carry out the study, the expectancy disconfirmation theory was used. This model was chosen above others because it applies to healthcare facilities and focusses majorly on customers’ satisfaction and has four constructs in which satisfaction is the main dependent construct.

The results of this study, hopefully, will guide efforts to raise the standard of labour and postpartum care in Nigeria and improve the quality of care being provided to pregnant women during antenatal, intrapartum and postpartum period in Nigeria.

## Research methods and design

### Study design

The study was a qualitative research of exploratory descriptive design carried out in Ogbomoso, Oyo State, Nigeria, using in-depth interviews. This design is suitable for this study because it has capability to explore factors that influence and interact with a phenomenon and eventually define the problem in question. The key principles applied include contextual understanding, open-ended questions and thick description of the phenomenon studied. Open-ended questions were designed to account for such flexibility in line with the focus of the study.

### Setting

Ogbomoso is a town with an estimated population of 454 690 people in the 2006 Nigerian Population Census.^20^ In addition to being one of the most populous cities in Nigeria, it is the second largest in Oyo State.

The study was conducted in the postnatal, family planning and infant welfare clinics of the Ladoke Akintola University Teaching Hospital, a government tertiary teaching hospital that provides obstetric care and receives referrals from the environ; the Bowen University Teaching Hospital, a faith-based tertiary institution; and the State Hospital, a government-owned secondary care level hospital run by Oyo State Hospital Management Board, all located in Ogbomoso. The town has a rich cultural heritage deeply rooted in Yoruba traditions. The primary language spoken in Ogbomoso is Yoruba, although many residents also speak English and other Nigerian languages because of its multiethnic nature. Traditional family values and customs play a crucial role in the social fabric.

### Study population and sampling strategy

Using purposive sampling method, consenting multiparous (2–4 children) women who were 1–6 weeks postnatal and had their antenatal care and delivery in the selected facilities were recruited. Those who had caesarean section and labour or delivery complications were excluded from the study. Purposive sampling was based on the judgement of the researchers regarding subjects and objects that are representative of the phenomenon being studied. The researchers intentionally drew a sample from the population that had the criteria expected in the participant; therefore, the members of the population do not have equal chance of being selected. This method was chosen for this study in order to enable the researchers have a diverse range of opinions and views, which will enhance credibility of the findings. The sample size for the study was 17 participants based on saturation. The clients who consented were selected for interview at an agreed time and venue. Saturation was reached at the 14th participant; however, three more participants were further interviewed to affirm the saturation.

### Data collection

Between October and November 2020, the first author assisted by two other members of the research team conducted and recorded an audiotape in-depth interview with postnatal women from selected health facilities within Ogbomoso. The date, time and venue of the interview were decided by the participants. Prior to the interview, the researchers explained the study’s objective and procedures to the participants and obtained their consent by having them sign a consent form. A semi-structured interview guide was used to conduct a face-to-face interview to allow participants express their views freely and enable researchers seek clarifications using probes. The interviews lasted 45 min – 60 min and the conversation was focused on factors influencing maternal satisfaction with labour care. The interviews were recorded with an audio recorder with permission from the participants. Field notes were also taken to record observations on non-verbal communication. Recorded interviews were transcribed verbatim and reviewed against the audio files and documented field notes for accuracy and clarifications from the participants. The study instrument was piloted at a primary health care facility.

In order to ensure the trustworthiness of the study, we implemented various approaches such as fostering strong interpersonal connection, building trust, triangulation of data gathering by examining the phenomenon from different perspectives, conducting member checks by seeking feedback from participants to ensure interpretations reflect their perspectives and performing a dependability audit. For the audit, a detailed record of the research process was kept and an external independent peer reviewed the process and the collected materials to ensure steps and decisions taken were logical and consistent.

### Reflexivity statement

Through reflexivity and bracketing, the researcher was able to guard her own biases, assumptions, opinion, beliefs and presupposition that might want to influence the study. This was carried out by maintaining a reflexive journal during the research process, keeping notes and documentation of daily introspections, which is beneficial and pertinent during the period of study. The researcher presented findings that emerged from the data as a true reflection of the perspectives of the postnatal women about their satisfaction with labour care services. The responses of the participants were recorded, transcribed verbatim and the themes that emerged were supported by direct quotes from the participants. In addition, the data collected and analysis were presented to the participants in order to ensure whether the narratives correlate and reflect their true experiences or not.

### Data management

Data collected during the interview were protected in order to maintain confidentiality of the participants. Each interviewee was given a code based on the facility of recruitment and pseudonyms were used to replace the codes after each interview. Participants’ recorded and transcribed interviews were saved in folders. The audiotape recordings, transcript of the interviews and field notes were kept in a safe locker separate from the demographic information and consent forms and made accessible to only the researchers. The raw data, stored in an external drive in order to guard against data loss, would be kept safe for a period of 5 years.

### Data analysis

Qualitative data analysis was performed concurrently and in stages after each interview for accuracy and clarification from the participants. The transcripts were examined for patterns or contrast ideas that emerged. Similar thoughts and words within the data were combined to develop a theme while the related themes were merged to form categories. Codes were assigned to the data by going through the responses of the participants and labelling them. Frequent themes were identified and linked together with the codes. The data were content analysed and the relationship between the responses was explored. The field notes were used to support the themes (such as quotations from the participants). All the identified formed categories were coded with subheadings and kept in a file, and then each new themes and categories identified afterward were added to the file. The process was continued until all the transcripts were exhausted. The relationship between the themes and categories was further analysed and grouped into major categories. Finally, tentative conclusion was drawn for the themes and categories identified to depict the perspectives of the postnatal mothers on the factors that influence satisfaction with labour care services. Atlas-Ti software version 9.0 was used to aid data analysis.

### Ethical considerations

Ethical clearance to conduct this study was obtained from the Bowen University Teaching Hospital, Ogbomoso, Bowen University Teaching Hospital Research Ethics Committee (No. BUTH/REC-090), before data collection was carried out. In addition, approval was also given by the unit heads in the facilities selected.

## Results

### Demographic characteristics

A total number of 17 postnatal women aged 25–43 years who were married and from the Yoruba ethnic group were recruited from the three major health facilities in Ogbomoso for the study. Two of the study participants were Muslims while the others were Christians. Almost all the participants (12) indicated that they resided in urban areas with only 5 being rural dwellers. Majority of the participants (16) affirmed that their pregnancies were planned while only one was accidental. They all delivered their babies in the respective health facilities where they had registered for their antenatal care, and their parity was from two to four. The information further revealed that majority of the participants (14) were literate and their occupation included: teaching (3), trading (4), fashion designing (2), catering (2) and clerical work (1), among others. Findings generated from the demographic data of the respondents revealed that academic qualification, occupation and other factors related to the background profile of the respondents may not likely inform the choice of health facility.

### Factors influencing maternal satisfaction with labour care services

Various factors could be deduced from the responses of the postnatal women that may influence the women’s satisfaction with labour care, which include the choice of health facility, cost effectiveness and availability of essential supplies, competent and respectful healthcare providers, environment of the facility and privacy, emergency preparedness and provision of needed information and education post-natal. It was discovered that the choice of facility has a great link with previous reputable services rendered by such facility. The acceptability of the labour care services led many participants to consider subsequent utilisation. The choice of facility was also not unrelated to recommendations by friends and significant others. Some of the responses of the interviewees in relation to the identified factors were as discussed in the following sections.

### Choice of healthcare facility

The women registered in the facilities for different reasons. P chose the facility where she delivered because she is a member of staff of the facility and she felt:

‘… I should be able to benefit from good health care.’ (P11, age 38, F)

Her statement confirmed that the health facility has been rendering excellent labour care service, which motivated her to register there. Another participant who had used the facility before for delivery mentioned that:

‘… [*D*]uring my previous pregnancy, that was my first born, I use this hospital and I enjoyed all the care then.’ (P17, age 42, F)

So she was motivated to patronise the place again.

This was also reported by another participant from the private health facility. She said:

‘I know them to be very good and skilled in addition to that, I had my first baby here and the midwife that took care of my delivery and my baby then was very excellent which was unlike what happened this time. She was very good, kind and skilled, so I felt definitely I needed to deliver there again.’ (P2, age 32, F)

### Cost effectiveness and availability of essential drugs

Most women were propelled to access a particular facility over and over again when the cost is affordable and the essential supplies are made available within the vicinity of the hospital. Most women utilise the public health facility because of the cheap cost of care and effective drugs as mentioned by the participants. A woman decided to register at the facility where she delivered her baby. She said:

‘[*A*]ctually, I choose the facility because it’s a government hospital, they usually have effective and essential drugs and the care is cost effective.’ (P17, 42, F)

### Presence of respectful and competent healthcare providers

Interviewees were satisfied with healthcare providers and attention they received. N said:

‘[*W*]hen I was about to deliver, there were four doctors present and midwives were also in attendance and I was impressed with that.’ (P08, age 33, F)

A participant from Ladoke Akintola Teaching Hospital (LTH) sharing her experience with the health workers said:

‘Ooh they are really good…. There is one particular doctor like that, I can’t remember her name, she was really there, she was really there for me. Any time I called her, she came to attend to me and said “just be praying, you will deliver very soon. They really tried”.’ (P12, age 30, F)

Glory, another participant, also reported that the care providers were well behaved:

‘The nurses attended to me well and showed good care. Their utterances were not discouraging as in other places. They spoke encouraging words to calm me down and reassure me.’ (P16, age 40, F)

S from LTH commended the comprehensive care received:

‘The necessary medical personnel were around. Also, when a paediatrician was needed at the point of my delivery, a consult was sent and the paediatrician was on ground to check the baby.’ (P09, age 35, F)

The midwives were also commended by another participant; she was satisfied with the way the midwives attended to her. In her words:

‘[*T*]hey were by my side. They gave me both psychological and emotional support and all that made me relaxed and also made me to feel at home.’ (P15, age 29. F)

The participants further revealed their satisfaction with the expertise demonstrated by the healthcare providers. Excerpt from a postnatal woman was:

‘[*T*]hey were excellent, they helped to suck my baby who had a breathing difficulty and they did it well … I love the way they rupture membranes to hasten labour. I noticed this was done in my previous labours and it worked well.’ (P16, age 40, F)

SL expressed her satisfaction with the efforts and competence of the healthcare providers:

‘I was most satisfied with the concern shown during the last stage of my labour. They put in a lot of efforts to provide necessary care to me.’ (P15, age 29, F)

### Presence of conducive environment for delivery and privacy

The cleanliness of the environment, provision of bed nets and window nets, and a good delivery couch were also factors that informed the satisfaction of the patients during delivery. A respondent from the State Hospital said:

‘[*T*]he environment was neat and quiet. It was okay … I was expecting power supply all through and it was so. I was also expecting a clean environment. The toilet was clean as well as the labour room and, beddings. The place was free of mosquitoes as against my expectation based on what I had been told initially.’ (P15, age 29, F)

S from LTH commended the privacy received:

‘I was given my privacy and was not exposed to anyone. It was only my husband and the necessary medical personnel that were around.’ (P09, age 35, F)

### Emergency preparedness plan

Participants from the private health facility claimed that the preparedness for labour was outstanding. A woman from the facility said:

‘[*E*]mergency care was given without delay. When my baby delayed in breathing, oxygen was immediately administered and the baby picked up thereafter.’ (P01, age 25, F)

### Information and education post-delivery

The women were satisfied with the counselling and instructions given to them about the care of their babies, immunisation and drugs.

A postnatal woman explained thus:

‘[*T*]he nurse talked to me about how to care for my baby and told me to come back for postnatal assessment, and family planning, then … immunisation for my baby.’ (P08, age 33, F)

‘They said I should be breastfeeding my baby very well. They educated me about family planning, immunization and so many things … (P12, age 30, F)

The communication of the health workers was also satisfying to the postnatal women. They explained that the health workers communicated both in the official language and the local language for the purpose of information clarity.

‘They also communicated with us in the language we understood; they spoke both Yoruba and English.’ (P03, age 38, F)

Supporting this, a participant commented:

‘[*T*]he health personnel communicated well and they ensured one understood what they were saying, so the communication was very good.’ (P12, age 30, F)

J, another respondent, explained how she received clear and tender communication from the nurses:

‘[*T*]hey explained how we should take our drugs adequately; I was told to avoid the baby being carried by many people and practice exclusive breastfeeding. I was also told to come for immunization on a regular basis and I was educated on family planning.’ (P10, age 32, F)

## Discussion

The study identified that various factors can influence maternal satisfaction with labour and childbirth positively. They include the following: (1) the choice of health facility, (2) cost effectiveness and availability of needed supplies, (3) respectful and competent care providers, (4) conducive environment, (5) ensuring privacy, (6) emergency preparedness and provision of needed information and (7) education post-natal. Studies across Ethiopia on maternal satisfaction with labour care service corroborated the findings that various factors can influence maternal satisfaction. The Ethiopian studies revealed that satisfaction of the mothers was predicted by a number of antenatal visits, non-payment for drugs and supplies, prompt attention to patients’ need, assurance of privacy during maternal examination and management of labour pains.^21,22,23,24^

The choice of facility has a great link with previous reputable services rendered by such facility.^25^ The acceptability of the labour care services received previously led many women in this study to consider subsequent utilisation of the facility in which they delivered. The choice of facility is also not unrelated to recommendations by friends and significant others.

Most women in this study were propelled to access care in a particular facility over and over again when the cost was affordable and essential supplies were made available within the vicinity of the hospital. The women affirmed that they utilised the public health facility because of the cheap cost of care and availability of effective drugs. These findings show that affordable cost of care and availability of supplies and drugs within the hospital are factors that influence maternal satisfaction and future utilisation of health facility. The findings are similar to that of a study from Ghana, which reported the emergence of affordable cost of care and access to care as factors that influence maternal satisfaction. It was further reported that because of the low resource context of developing countries, the poor will benefit from availability and access to medical care when affordable care is provided.^26^

The cost of labour and childbirth services seems to be a factor that influences women’s satisfaction. Most women from the public secondary health facility reported being satisfied. The reason may be that women pay lesser fee for their care there. This finding is consistent with a similar study in Ethiopia, which stated that women who paid less for labour care services reported higher satisfaction.^27^ Contrary to this finding, however, another study in Kenya revealed that the cost of care is not a factor responsible for women’s satisfaction with labour care.^28^ In Nigeria, women are made to pay for their care from antenatal to the point of delivery at the public secondary and tertiary healthcare facilities, except for the primary health centres where the cost is heavily subsidised by the government.

Furthermore, this study identified the presence of a respectful and skilful care provider as one of the factors that influenced maternal satisfaction with labour and delivery care. The care providers who attended to the women in labour were knowledgeable about maternity care and this enhanced their skilfulness in rendering their services. It was affirmed by the postnatal women that most of the healthcare providers knew the appropriate action to take in any given situation. This finding is similar to previous studies among women in health facilities of Ghana and Guinea, as well as systematic review of studies regarding maternity care satisfaction, which revealed that professional competence and respect for women along with attractive physical infrastructure were predictors of maternal satisfaction.^29,30,31^

The presence of a conducive environment for delivery is another factor that can influence maternal satisfaction. Cleanliness of the environment, provision of bed nets and window nets, and the availability of a good delivery couch were also factors that informed the satisfaction of the patients during delivery. The findings are in line with a previous study, which confirms that physical environment is significant to positive assessment of women’s satisfaction with regard to labour and child birth.^17^

A closely related factor to physical environment identified that appears to influence maternal satisfaction among the postnatal women in this study is ensuring privacy, which entails nursing the women in a private labour room or having minimal number of trainees involved in the care of the woman. In this study, most women who received maximum privacy reported being satisfied with labour care compared with those women who experienced a lack of privacy. This finding is consistent with that of a study carried out in the Amhara region of Ethiopia where a lack of privacy during childbirth services was a source of dissatisfaction for women who sought such services.^27^ In addition, in this study, the postnatal women who complained about inadequate privacy had high educational background. Studies across Ghana, Gambia and Nigeria showed similar findings that educational status of women determined the women’s satisfaction with labour and childbirth with reference to privacy.^26,32,33^

The study by Srivastava et al. also highlighted conducive physical environment for delivery and privacy, as factors that influence maternal satisfaction.^17^ Various studies carried out on maternal satisfaction with delivery services among postnatal women in government hospitals in India, Nepal and Malaysia corroborate the result in this study.^7,34,35^

Postnatal women in this study were also satisfied with the availability of emergency care as well as with the information and instructions given to them about the care of their babies, immunisation and drugs. Women from the private health facility claimed that the preparedness for labour was outstanding. Emergency preparedness during labour is essential and can influence maternal satisfaction.

The participants also commented on the style of communication of the health workers. The care providers’ means of communication was the official language and the local language for the purpose of information clarity. They expressed satisfaction with the manner and mode of counselling and instructions given to them about the care of their babies, immunisation and drugs. This is similar to what was reported in a study carried out by Ferrer and colleagues, which stated that establishment of communication with the women in labour by professionals led to satisfaction. Also, providing opportunity of adequate information regarding the delivery during the labour and postnatal periods is a satisfying experience.^36^

## Conclusion

This study showed that the factors that influence maternal satisfaction were related to cost of care, competence of care provider, confidential and dignified care and availability of supplies. Government and hospital management should address these issues to enhance maternal satisfaction with labour care services.

### Recommendations

The study identified the cost of labour and childbirth services to be a factor that influences maternal satisfaction; therefore, government should establish community health insurance scheme that will improve healthcare financing and thus take off the burden of out-of-pocket payment for delivery services. Also, supplies and commodities should be made available and accessible at affordable cost. Furthermore, during labour and delivery, the number of trainees involved in the care of patients should be limited to ensure privacy.
